# 4,4′-Bis(2-methoxy­lstyr­yl)biphen­yl

**DOI:** 10.1107/S1600536809026920

**Published:** 2009-07-18

**Authors:** Yu-Feng Li, Fang-Fang Jian

**Affiliations:** a Microscale Science Institute, Department of Chemistry and Chemical Engineering, Weifang University, Weifang 261061, People’s Republic of China; b Microscale Science Institute, Weifang University, Weifang 261061, People’s Republic of China

## Abstract

The title compound, C_30_H_26_O_2_, was prepared by the reaction of a Wittig reagent and 2-methoxy­benzaldehyde. The mol­ecule lies about an inversion centre located at the midpoint of the C—C bond between the inner benzene rings. The crystal structure is stabilized by C—H⋯π inter­actions.

## Related literature

For the optical properties of ethyl­ene biphenyls, see: Song *et al.* (2003 ▶). For comparative bond lengths, see: Trueblood *et al.* (1982 ▶).
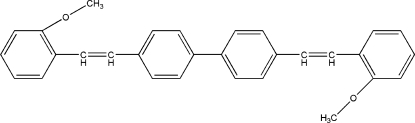


## Experimental

### 

#### Crystal data

C_30_H_26_O_2_
*M* *_r_* = 418.51
Monoclinic, 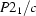

*a* = 15.499 (3) Å
*b* = 5.5050 (11) Å
*c* = 13.445 (3) Å
β = 98.61 (3)°
*V* = 1134.2 (4) Å^3^
*Z* = 2
Mo *K*α radiation
μ = 0.08 mm^−1^
*T* = 293 K
0.25 × 0.20 × 0.18 mm

#### Data collection

Bruker SMART CCD area-detector diffractometer
Absorption correction: none
2541 measured reflections
2431 independent reflections
1144 reflections with *I* > 2σ(*I*)
*R*_int_ = 0.031

#### Refinement

*R*[*F* ^2^ > 2σ(*F* ^2^)] = 0.065
*wR*(*F* ^2^) = 0.218
*S* = 1.01
2431 reflections
146 parameters
H-atom parameters constrained
Δρ_max_ = 0.22 e Å^−3^
Δρ_min_ = −0.18 e Å^−3^

### 

Data collection: *SMART* (Bruker, 1997 ▶); cell refinement: *SAINT* (Bruker, 1997 ▶); data reduction: *SAINT*; program(s) used to solve structure: *SHELXS97* (Sheldrick, 2008 ▶); program(s) used to refine structure: *SHELXL97* (Sheldrick, 2008 ▶); molecular graphics: *SHELXTL* (Sheldrick, 2008 ▶); software used to prepare material for publication: *SHELXTL*.

## Supplementary Material

Crystal structure: contains datablocks global, I. DOI: 10.1107/S1600536809026920/at2831sup1.cif

Structure factors: contains datablocks I. DOI: 10.1107/S1600536809026920/at2831Isup2.hkl

Additional supplementary materials:  crystallographic information; 3D view; checkCIF report

## Figures and Tables

**Table 1:** Hydrogen-bond geometry (Å, °)

*D*—H⋯*A*	*D*—H	H⋯*A*	*D*⋯*A*	*D*—H⋯*A*
C1—H1*C*⋯*Cg*1^i^	0.96	2.76	3.611 (3)	149
C15—H15*A*⋯*Cg*2^ii^	0.93	2.91	3.643 (3)	137

Symmetry codes: (i) 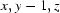; (ii) 
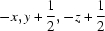
. *Cg*1 and *Cg*2 are the centroids of the C2–C7 and C10–C15 rings,respectively.

**Fig. 1.**
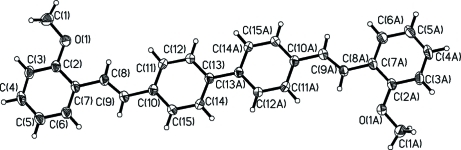
The structure of the title compound showing 30% probability displacement ellipsoids and the atom-numbering scheme.

## supplementary crystallographic information

### Comment

Ethylene biphenyl have received considerable attention in the literature. They are attractive from several points of view, such as the optics characteristic. (Song *et al.*, 2003). As part of our search for new ethylene biphenyl compounds we synthesized the title compound (I), and describe its structure here.

As shown in Fig. 1, the molecule has an inversion centre lied on the midpoint of the C—C bond between the inner benzene rings. The C8—C9 bond length of 1.314 (4)Å is comparable with C—C double bond [1.336 (2) Å] reported (Trueblood *et al.*, 1982).

In the structure, there is no classcial hydrogen bonds. The crystal structure is stabilized by C—H···π interactions (Table 1).

### Experimental

A mixture of the Wittig-reagent (0.1 mol), and 2-methoxybenzaldehyde (0.2 mol) was stirred in refluxing *N*,*N*-dimethylformamide (20 mL) for 4 h to afford the title compound (0.084 mol, yield 84%). Single crystals suitable for X-ray measurements were obtained by recrystallization from ethanol at room temperature.

### Refinement

H atoms were fixed geometrically and allowed to ride on their attached atoms, with C—H = 0.93 - 0.96 Å, and with *U*_iso_(H) =1.2 or 1.5*U*_eq_(C).

### Crystal data

**Table:**

C_30_H_26_O_2_	*F*(000) = 444
*M**_r_* = 418.51	*D*_x_ = 1.225 Mg m^−^^3^
Monoclinic, *P*2_1_/*c*	Mo *K*α radiation, λ = 0.71073 Å
Hall symbol: -P 2ybc	Cell parameters from 987 reflections
*a* = 15.499 (3) Å	θ = 2.2–27.5°
*b* = 5.5050 (11) Å	µ = 0.08 mm^−^^1^
*c* = 13.445 (3) Å	*T* = 293 K
β = 98.61 (3)°	Block, yellow
*V* = 1134.2 (4) Å^3^	0.25 × 0.20 × 0.18 mm
*Z* = 2	

### Data collection

**Table:**

Bruker SMART CCD area-detector diffractometer	1144 reflections with *I* > 2σ(*I*)
Radiation source: fine-focus sealed tube	*R*_int_ = 0.031
graphite	θ_max_ = 27.0°, θ_min_ = 1.3°
φ and ω scans	*h* = −18→18
2541 measured reflections	*k* = −6→0
2431 independent reflections	*l* = −16→0

### Refinement

**Table:**

Refinement on *F*^2^	Secondary atom site location: difference Fourier map
Least-squares matrix: full	Hydrogen site location: inferred from neighbouring sites
*R*[*F*^2^ > 2σ(*F*^2^)] = 0.065	H-atom parameters constrained
*wR*(*F*^2^) = 0.218	*w* = 1/[σ^2^(*F*_o_^2^) + (0.1165*P*)^2^] where *P* = (*F*_o_^2^ + 2*F*_c_^2^)/3
*S* = 1.01	(Δ/σ)_max_ < 0.001
2431 reflections	Δρ_max_ = 0.22 e Å^−^^3^
146 parameters	Δρ_min_ = −0.18 e Å^−^^3^
0 restraints	Extinction correction: *SHELXL97* (Sheldrick, 2008), Fc^*^=kFc[1+0.001xFc^2^λ^3^/sin(2θ)]^-1/4^
Primary atom site location: structure-invariant direct methods	Extinction coefficient: 0.045 (8)

### Special details

**Table:**

Geometry. All e.s.d.'s (except the e.s.d. in the dihedral angle between two l.s. planes) are estimated using the full covariance matrix. The cell e.s.d.'s are taken into account individually in the estimation of e.s.d.'s in distances, angles and torsion angles; correlations between e.s.d.'s in cell parameters are only used when they are defined by crystal symmetry. An approximate (isotropic) treatment of cell e.s.d.'s is used for estimating e.s.d.'s involving l.s. planes.
Refinement. Refinement of *F*^2^ against ALL reflections. The weighted *R*-factor *wR* and goodness of fit *S* are based on *F*^2^, conventional *R*-factors *R* are based on *F*, with *F* set to zero for negative *F*^2^. The threshold expression of *F*^2^ > σ(*F*^2^) is used only for calculating *R*-factors(gt) *etc*. and is not relevant to the choice of reflections for refinement. *R*-factors based on *F*^2^ are statistically about twice as large as those based on *F*, and *R*- factors based on ALL data will be even larger.

### Fractional atomic coordinates and isotropic or equivalent isotropic displacement parameters (Å^2^)

**Table:**

	*x*	*y*	*z*	*U*_iso_*/*U*_eq_	
O1	0.37628 (14)	0.1134 (4)	0.17470 (14)	0.0684 (7)	
C1	0.4420 (2)	−0.0626 (6)	0.1680 (2)	0.0738 (11)	
H1A	0.4491	−0.1627	0.2271	0.111*	
H1B	0.4961	0.0178	0.1627	0.111*	
H1C	0.4254	−0.1619	0.1096	0.111*	
C2	0.35652 (18)	0.2759 (5)	0.09673 (19)	0.0493 (7)	
C3	0.3997 (2)	0.2830 (6)	0.0136 (2)	0.0602 (9)	
H3A	0.4442	0.1727	0.0082	0.072*	
C4	0.3770 (2)	0.4529 (7)	−0.0611 (2)	0.0665 (10)	
H4A	0.4066	0.4568	−0.1164	0.080*	
C5	0.3114 (2)	0.6160 (6)	−0.0545 (2)	0.0625 (9)	
H5A	0.2963	0.7302	−0.1051	0.075*	
C6	0.26746 (19)	0.6097 (6)	0.0283 (2)	0.0565 (8)	
H6A	0.2233	0.7216	0.0327	0.068*	
C7	0.28816 (16)	0.4391 (5)	0.10519 (19)	0.0456 (7)	
C8	0.24047 (17)	0.4232 (5)	0.19277 (19)	0.0497 (8)	
H8A	0.2537	0.2911	0.2354	0.060*	
C9	0.18161 (18)	0.5755 (5)	0.2166 (2)	0.0493 (8)	
H9A	0.1705	0.7120	0.1759	0.059*	
C10	0.13109 (17)	0.5531 (5)	0.30137 (19)	0.0434 (7)	
C11	0.1426 (2)	0.3639 (6)	0.3705 (2)	0.0616 (9)	
H11A	0.1851	0.2472	0.3654	0.074*	
C12	0.0920 (2)	0.3457 (5)	0.4468 (2)	0.0615 (9)	
H12A	0.1016	0.2159	0.4914	0.074*	
C13	0.02809 (16)	0.5122 (5)	0.45942 (19)	0.0406 (6)	
C14	0.0180 (2)	0.7035 (5)	0.3911 (2)	0.0556 (8)	
H14A	−0.0239	0.8216	0.3970	0.067*	
C15	0.0683 (2)	0.7237 (6)	0.3145 (2)	0.0576 (9)	
H15A	0.0596	0.8553	0.2707	0.069*	

### Atomic displacement parameters (Å^2^)

**Table:**

	*U*^11^	*U*^22^	*U*^33^	*U*^12^	*U*^13^	*U*^23^
O1	0.0824 (16)	0.0795 (15)	0.0477 (12)	0.0305 (13)	0.0238 (11)	0.0099 (12)
C1	0.083 (2)	0.081 (3)	0.057 (2)	0.027 (2)	0.0114 (17)	−0.0030 (18)
C2	0.0517 (16)	0.0613 (18)	0.0365 (15)	−0.0004 (15)	0.0114 (12)	−0.0031 (14)
C3	0.0571 (18)	0.082 (2)	0.0457 (17)	0.0071 (17)	0.0228 (15)	−0.0038 (17)
C4	0.0594 (19)	0.104 (3)	0.0409 (17)	−0.0055 (19)	0.0235 (14)	0.0008 (18)
C5	0.0603 (19)	0.089 (2)	0.0393 (16)	−0.0015 (19)	0.0110 (14)	0.0128 (17)
C6	0.0491 (16)	0.075 (2)	0.0465 (16)	0.0034 (16)	0.0091 (13)	0.0075 (16)
C7	0.0404 (15)	0.063 (2)	0.0350 (14)	−0.0034 (13)	0.0107 (11)	−0.0011 (13)
C8	0.0475 (16)	0.065 (2)	0.0393 (15)	0.0066 (15)	0.0136 (12)	0.0041 (14)
C9	0.0523 (17)	0.0556 (19)	0.0423 (15)	−0.0007 (15)	0.0142 (12)	0.0004 (13)
C10	0.0411 (15)	0.0501 (17)	0.0397 (15)	−0.0015 (13)	0.0081 (12)	−0.0064 (13)
C11	0.0591 (18)	0.065 (2)	0.067 (2)	0.0249 (16)	0.0306 (16)	0.0138 (17)
C12	0.067 (2)	0.063 (2)	0.0608 (19)	0.0243 (17)	0.0318 (16)	0.0209 (16)
C13	0.0403 (14)	0.0462 (15)	0.0357 (13)	−0.0002 (12)	0.0078 (10)	−0.0044 (12)
C14	0.0585 (18)	0.0579 (19)	0.0554 (17)	0.0198 (15)	0.0248 (14)	0.0066 (15)
C15	0.0662 (19)	0.062 (2)	0.0490 (16)	0.0172 (16)	0.0235 (15)	0.0154 (15)

### Geometric parameters (Å, °)

**Table:**

O1—C2	1.377 (3)	C8—C9	1.314 (4)
O1—C1	1.419 (4)	C8—H8A	0.9300
C1—H1A	0.9600	C9—C10	1.482 (4)
C1—H1B	0.9600	C9—H9A	0.9300
C1—H1C	0.9600	C10—C15	1.383 (4)
C2—C3	1.386 (4)	C10—C11	1.389 (4)
C2—C7	1.407 (4)	C11—C12	1.386 (4)
C3—C4	1.379 (4)	C11—H11A	0.9300
C3—H3A	0.9300	C12—C13	1.379 (4)
C4—C5	1.370 (4)	C12—H12A	0.9300
C4—H4A	0.9300	C13—C14	1.391 (4)
C5—C6	1.390 (4)	C13—C13^i^	1.500 (5)
C5—H5A	0.9300	C14—C15	1.387 (4)
C6—C7	1.398 (4)	C14—H14A	0.9300
C6—H6A	0.9300	C15—H15A	0.9300
C7—C8	1.484 (3)		
			
C2—O1—C1	118.4 (2)	C9—C8—C7	127.1 (3)
O1—C1—H1A	109.5	C9—C8—H8A	116.5
O1—C1—H1B	109.5	C7—C8—H8A	116.5
H1A—C1—H1B	109.5	C8—C9—C10	126.9 (3)
O1—C1—H1C	109.5	C8—C9—H9A	116.5
H1A—C1—H1C	109.5	C10—C9—H9A	116.5
H1B—C1—H1C	109.5	C15—C10—C11	116.5 (2)
O1—C2—C3	123.6 (3)	C15—C10—C9	120.3 (3)
O1—C2—C7	116.0 (2)	C11—C10—C9	123.2 (2)
C3—C2—C7	120.5 (3)	C12—C11—C10	121.3 (3)
C4—C3—C2	120.4 (3)	C12—C11—H11A	119.4
C4—C3—H3A	119.8	C10—C11—H11A	119.4
C2—C3—H3A	119.8	C13—C12—C11	122.7 (3)
C5—C4—C3	120.5 (3)	C13—C12—H12A	118.7
C5—C4—H4A	119.7	C11—C12—H12A	118.7
C3—C4—H4A	119.7	C12—C13—C14	115.7 (2)
C4—C5—C6	119.5 (3)	C12—C13—C13^i^	122.4 (3)
C4—C5—H5A	120.2	C14—C13—C13^i^	121.8 (3)
C6—C5—H5A	120.2	C15—C14—C13	122.1 (3)
C5—C6—C7	121.6 (3)	C15—C14—H14A	119.0
C5—C6—H6A	119.2	C13—C14—H14A	119.0
C7—C6—H6A	119.2	C10—C15—C14	121.7 (3)
C6—C7—C2	117.5 (2)	C10—C15—H15A	119.2
C6—C7—C8	122.7 (3)	C14—C15—H15A	119.2
C2—C7—C8	119.8 (3)		

Symmetry codes: (i) −*x*, −*y*+1, −*z*+1.

### Hydrogen-bond geometry (Å, °)

**Table:**

*D*—H···*A*	*D*—H	H···*A*	*D*···*A*	*D*—H···*A*
C1—H1C···Cg1^ii^	0.96	2.76	3.611 (3)	149
C15—H15A···Cg2^iii^	0.93	2.91	3.643 (3)	137

Symmetry codes: (ii) *x*, *y*−1, *z*; (iii) −*x*, *y*+1/2, −*z*+1/2.
